# The economics of secession: a review of legal, theoretical, and empirical aspects

**DOI:** 10.1186/s41937-017-0015-6

**Published:** 2018-04-03

**Authors:** Thierry Madiès, Grégoire Rota-Grasiozi, Jean-Pierre Tranchant, Cyril Trépier

**Affiliations:** 1 Department of Economics, University of Fribourg, Fribourg, Switzerland; 2 University of Clermont Auvergne, CNRS, CERDI, Clermont-Ferrand, France; 3 Institute of Development Studies, University of Sussex, Brighton, UK; 4 CRAG, University of Paris 8 Saint-Denis, Paris, France

## Abstract

The aim of this paper is to present a review of the legal, theoretical, and empirical aspects of secessions from an economic perspective. This survey provides interesting insights into recent events such as the Brexit and the threat of secession made for instance by Scotland and Catalonia. International law does not grant a general right to secede, nor does it forbid secession. Furthermore, there are several modalities of secessions, which turn out to be important for new states that want to get an international recognition. For its part, the economic theory shows that the decision for a region to remain in a country (or a union) or to secede eventually results from a trade-off between the benefits of being part of a large country, on the one hand, and the costs often associated to more heterogeneity, on the other hand. The latter are generally more important for those regions which are “far away” from the central (federal) government. Empirical literature confirms the importance of these trade-offs and shows that decentralization may be effective to accommodate secessionist conflicts only if certain conditions are fulfilled.

## Introduction

Neither the binding referendum held in Scotland on 18 December 2014, nor the unilateral declaration of independence in Catalonia on 27 October 2017 created a new state. The former gave victory to the “No”; the latter, less well prepared than the Scottish referendum, led to the destitution of the Catalan government and to new regional elections to be held on 21 December 2017. Despite their differences, both events remind us that states are not immutable. Separatist forces are still present in certain EU member states. In their seminal and influential paper about the breakup of nations, Bolton and Roland (1997) state that “the entire map of Europe, from the Atlantic Coast to the Urals, is being redrawn and issues of separation, unification, and the redrawing of borders are yet again at the forefront of European concerns. Many of the issues raised by this process are primarily of a political, cultural or linguistic nature. However, there are also economic considerations that bear on this problem”.

Switzerland has not escaped from secessionist desires. The most famous case is that of the Canton of Jura which was created on 1 January 1979 after having seceded from the Canton of Berne. Dominicé (2006) points out that “when for a specific matter there is no federal rule, international law is applied as a substitute […] In the case of the Canton of Jura, important questions were settled by reference to customary international law under the laws of State secession.”

Secessionism is obviously not only a threat to European states but is also a concern in a great number of developing and ethnically fragmented countries, where it takes often a violent form. Since World War II, the number of new sovereign states has dramatically increased from 51 in 1945 to 99 in 1960 and to 195 in 2017 mainly due to the decolonization process of the 1960s and the breakup of the Soviet Union and Yugoslavia in the 1990s. Sambanis et al. (2017) estimate the number of groups that have made separatist claims in 120 countries over the period 1945–2012 at 464, that is to say twice as much as the figures provided by former studies.

Widespread secessionism exists even in the face of high uncertainty about the economic impact of secessions. In fact, secessions may well be economically costly. Smaller countries face a steeper cost of providing public goods, and their small internal markets do not allow to reach maximum efficiency in the production of private goods (except if they enjoy the proximity to a large integrated market such as the European single market). Indeed, Reynaerts and Vanschoonbeek (2016) estimate that newly independent countries tend to grow more slowly than other comparable countries. The authors find, on average, that the cost of secession is equivalent to 20% of GDP per capita, and it remains strong in the long run. There seems to be, however, a considerable variation: Secessions seem to have been particularly costly among ex-USSR Republics and French colonies but to have been much less costly in ex-Yugoslavia and ex-Czechoslovakia.

The fact that so many regions seek independence—even when secession is associated with economic costs—suggests that there are strong non-economic benefits of secession. Economic theory assumes that the greater homogeneity (geographically, culturally, linguistically, and otherwise) that characterizes smaller countries explains the importance of non-economic benefits. Regions that are geographically and culturally distant from the center may not receive much public goods and/or may have sharply distinct preferences on the type of public good they want. Some proponents of Scottish independence argued that secession would indeed be desirable as the preferences of Scots on social policy are very different from those of the rest of the UK. Desmet et al. (2011) argue that the breakup of the ex-Yugoslavia would not have happened for economic reasons alone and required a preference for cultural homogeneity.

Both political and fiscal decentralization have been widely used by central governments as a means to accommodate the claim of geographically concentrated groups and minorities for self-determination, with mixed results. This may be explained by three main reasons: The first one is that decentralization tends to be used by central governments to appease separatists, hence a seemingly positive relationship between centrifugal forces and decentralization. The second one is that subnational groups or entities that are granted greater autonomy might use the available resources in their hands to foster separatist tendencies. The third reason is that decentralization may have different meanings for central (federal) governments and subnational jurisdictions. Central governments very often want to retain power so that decentralization actually boils down to a simple deconcentration, i.e., responsibilities over taxes and public expenditures are actually transferred to a representative of the central government at the subnational level and not to locally elected officials (Dafflon and Madiès 2011).

The aim of this paper is to present a survey on secessions covering both legal issues and the main contributions of the economic literature, with a particular focus on the question as to whether decentralization fosters or hinders the desire to secede. More specifically, the paper is organized as follows. The first part deals with both international and EU law regarding the secession phenomenon. The second part presents the main contributions of the economic theory of secessions. The third part presents empirical evidence that confirms the main determinants of secessions identified by the theory. A more specific focus is put on ethnic conflicts and decentralization.

## The secession phenomenon as both the international and EU law regard it

We first present what the international law says about secessions. Then, we deal with the different modalities of separation and analyze some recent secession processes. Finally, we take a look at the EU law. It must be noticed that “international law does not grant sub-state entities a general right to secede from their parent states, nor does it prohibit secession” (Roethke 2011).^1^ Regarding the EU law, it must be remembered that the European Union has so far never experienced any internal secession in which the new state wanted to join the organization. Indeed, neither Algeria in 1962 when becoming independent from France nor Greenland in 1979 when obtaining home rule from Denmark wanted to be part of the European Economic Community. Besides, even if the European map has changed considerably since the collapse of Soviet Union in 1991, the numerous newly created states seceded before becoming EU member states.

### The procedures of secession under the international law

For newly created countries, obtaining a wide international recognition is not a mere formality. Indeed, international law recognizes the right to self-determination only in the cases of decolonization process and for groups discriminated against on the account of an ethnic minority status. The right to self-determination is protected by the Article 1.2 of the United Nations Charter signed in 1945, which reads: “To develop friendly relations among nations based on respect for the principle of equal rights and self-determination of peoples, and to take other appropriate measures to strengthen universal peace.” This general principle was strengthened later on when the United Nations General Assembly voted on 12 December 1960 the 1514 Resolution^2^ called “Declaration on the granting of independence to colonial countries and peoples” (Rosiere 2010). Once adopted, the resolution became the norm to interpret the right of people to self-determination in the European colonies. Yet, it appeared quite difficult to determine exactly the application field of this right, since other situations where “peripheries” consider themselves dominated by a “center” present some similarity with the situation in former European colonies: Siberia in Russia, Abkhazia in Georgia, Western Sahara in Morocco, and Tibet and Xinjiang in China for instance. The UN resolution 61/295 adopted on 13 September 2007 and entitled “Declaration on the Rights of Indigenous peoples” tends to extend the scope of implementation of the self-determination right to any place in the world and to enable any territory to claim this right when it asserts “the fundamental importance of the right to self-determination of all peoples, by virtue of which they freely determine their political status and freely pursue their economic, social, and cultural development”.^3^ More specifically, it may be argued that a subset of people within a country who would suffer from “serious breaches of fundamental human and civil rights” through the “abuse of ‘sovereign power’ could be recognized the right to separate from the ‘offending state” (Tancredi 2006).

Nevertheless, the international community sets some limits to the application of the self-determination right to avoid an avalanche of secession claims. One of these is the principle of respect for the state’s territorial integrity, since it can be understood as protecting both internal and external borders of a state.^4^ The previously mentioned UN 1514 Resolution of 12 December 1960 states in its article 6 that “any attempt aimed at the partial or total disruption of the national unity and the territorial integrity of a country is incompatible with the purposes and principles of the Charter of the United Nations”.

Yet, the United Nations has considered on several occasions that separation was a legitimate conflict resolution mode. It did so in Eastern Timor in 1999—where its intervention was necessary to get Indonesia to accept the outcome of the popular vote—in Bougainville in 2001 or in Southern Sudan in 2011. Those three cases show that self-determination has become a norm for nonviolent separations. In the case of Bougainville, which claims its separation from Papua New Guinea, the 2001 peace agreement includes a self-determination referendum, which should take place in 2019.

Each binding self-determination referendum requires determining who votes, how the vote must take place, and which territory is implied. Yet, the international recognition is crucial to determine the success or failure of a separation process.

### Different modalities of secession

The following table shows that three modalities of secession exist under the international law: An armed conflict (like in Croatia), a unilateral separation (Kosovo), and an agreement with the central state (Montenegro). These situations have in common that the desire for secession comes from one part of the initial territory. In turn, an unanimous will of separation, representing the whole population and territory of a state, provokes a scission. The initial state is dissolved in favor of several entities. A recent example of a scission is the separation of the Czech and Slovak Federal Republic into Czech Republic and Slovakia on 31 December 1992, which was negotiated by governments. We call successor state a state which has lost a part of its territory by secession but keeps existing under international law. Among others, it was the case of Pakistan after the secession of Bangladesh in 1971, of Ethiopia when Eritrea became independent in 1993, of Serbia after the separation of Montenegro in 2006, and of Sudan after the independence of South Sudan in 2011. Nevertheless, both a separation and a scission may provoke the dissolution of the initial state. Indeed, if after one or several secessions a state is regarded as substantially different from the initial state, it may lose its juridical personality and transform itself into a new state. As an illustration, we may recall that the Yugoslavian Federal Republic was not recognized as the continuator state after four out of six of its entities became independent.

Independence obtained through a unilateral declaration has very little chance, if at all, to be followed by a recognition from other sovereign states. Besides, the unilateral separation of a territory does not make it instantaneously independent without a negotiation with the central state. It implies that a separation of states requires two actors, or even three if the central state belongs to the European Union, to materialize. Hence, the interest of determining what the EU law says about independence inside the EU.

**Table:** 

A selection of secession processes since 1991^5^			
New state	Year	Context of secession	Modality of secession
Baltic states Lithuania, Latvia, Estonia	1990	The 3 Baltic states, which were Socialist Republics for 45 years, rejected the official vision of a “voluntary adhesion” to the USSR. They experienced a brief independence between 1918 and 1940. Independence was not claimed as secession, but to re-establish the “pre-existing state sovereignty confiscated by a neighbor power” according to international laws. August 23, 1989, a huge human chain connected the 3 Baltic capitals. The 3 Republics faced economic and military pressure from Moscow and boycotted the federal referendum of March 17, 1991, on a renovated Union.	Unilateral declarations (March 11, 1990, in Lithuania and August 21, 1991, for Latvia and Estonia). New Russian President B. Yeltsin recognized them on Sep. 6, 1991. September 17, 1991, the 3 Baltic states become UN member states. They joined EU in 2004.
Slovenia	1991	The richest province of Yugoslavia prepared independence before Croatia. At first, Slovenia called for a transformation of Yugoslavia into a confederation. The Parliament then voted constitutional amendments and a declaration of sovereignty. Citizens backed secession by a referendum in December 1990.	Declaration of independence from Yugoslavia on July 25, 1991. After an intervention of the Yugoslavian army and a 3-day conflict, a ceasefire was signed on June 28, 1991. Independence will was reasserted in October 1991 and backed by an arbitration commission of EEC. Germany recognized Slovenia as a state in December 1991; then, the other EU states did so in January 1992.
Croatia	1991	The second richest province of Yugoslavia held its first free elections in 1990, with a victory of a nationalist party, HDZ. In a referendum held in May 1991, 94% of Croatian voters backed independence. Croatian leaders made it clear they were inspired by Slovenia’s project of independence.	Declared independence from Yugoslavia on July 25, 1991. Serbs from Krajina soon backed by the federal army, attacked Croatia, triggering a war until December 1995. The EEC first rejected recognizing Croatia as a state, but Germany did so in December 1991. The UN intervened in March 1992.
Eritrea	1993	An independence movement against Ethiopia appeared in 1961 in Eritrea. In 1952, Eritrea was attached to the crown of Ethiopia as a federal state but became a province of Ethiopia by 1962. After 30 years of conflict, the main independence movement opened negotiations with the Ethiopian government in 1991, once General Mengistu’s regime was dissolved.	An official binding referendum on the independence of Eritrea was held in April 1993 with UN observer mission. Independence from Ethiopia received an overwhelming support (99.83% of voters) while the turnout reached 98.2% of registered voters.
Slovakia and Czech Republic	1993	Both formed the Czech and Slovak Federative Republic (April 23, 1990–December 31, 1992) created after the Czech and Slovak Socialist Republic (1948–1990) was dissolved. In June 1992, each republic held free elections, and then, Czech Prime Minister Vaclav Klaus and Slovakian Prime minister Vladimir Meciar opened negotiations to disband the federation.	Separation in 2 independent states was proclaimed on January 1, 1993. The Velvet divorce was a peaceful process but was initiated by both governments, not by the citizens. Both states joined the EU in 2004.
Quebec	1995	The question of independence was put twice to the vote of citizens of Quebec, a province of the federal state of Canada. After the 1980 official binding referendum in which secession had been rejected, a new one took place on October 30, 1995. The Supreme Court of Canada issued in 2000 the so-called law of clarity which set two conditions for a further independence referendum of a Canadian province: The question should refer to independence, and the result should “result on a clear expression of the will of the population.”	Independence from Canada was rejected (50.58% against, 49.42% in favor) as a result of a referendum with an unprecedented turnout: 93.25% of the electorate.
East Timor	2002	In this former colony of Portugal, several movements pro-independence appeared in 1974. One of them, the Fretilin, claimed the independence of East Timor on November 28, 1975. 9 days later, on December 7, 1975, Indonesia launched a military occupation of East Timor, an Indonesian province from July 1976. The conflict continued while several powers (USA, Australia, USSR) backed Indonesia against Timor. Once Suharto was forced to leave power in 1998, new President Habibie finally accepted in 1999 a UN-negotiated agreement including a short-term consultation on a wide autonomy project for East Timor.	The consultation took place on August 30, 1999, despite violence and intimidation against civilians from pro-Indonesian militias. The turnout reached 99.6% of registered voters, and 78.5% of them rejected autonomy, therefore backing independence. Heavy violence perpetrated after the vote by pro-Indonesian militias. President Habibie only accepted the result of the vote on October 19, 1999, after the intervention of a UN peace force. After a provisory administration by the United Nations (1999–2002) East Timor proclaimed its independence on May 2002.
Montenegro	2006	In Montenegro, there is a long-standing will of a peaceful independence from Serbia. In a previous referendum held in 1992, 95.96% of the population voted to remain in the federation. In 2000, while Serbia and Montenegro were already negotiating separation, the EU required a two-step process: an agreement on terms of separation and an official binding referendum.	Independence from Yugoslavia gained a 55.5% approval with a turnout of 86.5% on May 21 2006, binding referendum. EU had set a threshold of 55% of voters and a minimal turnout of 50% to validate the referendum. Independence was proclaimed on June 3, 2006.
Kosovo	2008	Having lost its autonomy status in 1989, the Serbian province initiated a peaceful fight to recover it. A war took place (1998–2001) despite international mediation (1998 and 1999). Kosovo is put under a provisory UN administration (MINUK) from 1999. UN special envoy Martti Ahtisaari issued on February 2, 2007, a proposition of resolution including “an independence under international control” for Kosovo. Both Serbia and Russia rejected it, maintaining the gridlock.	A unilateral declaration from the Kosovar Parliament on February 17, 2008, made the country an independent state. Belgrade denounced that declaration as illegal. A new UN mission is deployed all over Kosovo in December 2008. Seized by the Serbian government on that unilateral process of secession occurred on 17 February 2008, the Court of Justice in The Hague declared it conform to the international law. A normalization agreement firmed with Serbia on April 9 2013. 111 UN member states recognized Kosovo by 2016, but 5 EU states refuse to do so: Spain, Slovakia, Rumania, Cyprus, and Greece.
South Sudan	2011	Two civil wars (1955–1972 and 1983–2005) took place in Sudan. After the second one, a peace agreement signed in 2002 opened the way for a binding independence referendum in South Sudan within 6 years, on January 9, 2011. That referendum was held with international observers.	Independence from Sudan gained an overwhelming support at the January 2011 referendum: 98.83% of voters for “Yes”, and turnout reached 97.58% of registered voters, far beyond the required turnout of 60%. Independence proclaimed on July 9, 2011. Both civil wars in Sudan are responsible for at least 2.5 million deaths. A civil war broke out in December 2013.
Scotland	2014	A first binding referendum was held on September 18, 2014, after the Edinburgh agreement was signed on October 15, 2012, by British Prime Minister and Scottish First Minister.	Independence from UK was rejected as a result of an official binding referendum with a 84.59% turnout.

It follows from the above comments that two contradictory principles have been governing the United Nations toward secession claims: self-determination and states’ territorial integrity. In the absence of a precedent or immediate risk inside the EU when they were redacted, the European treaties do not dedicate a single line to internal secession.

### No explicit mention in the EU law

Without any precedent or immediate risk when these texts were debated, the European treaties do not say a word about internal secessions. Of course, some EU member states did experience a separation. It happened when Algeria became independent from France in 1962. More recently, Greenland took its autonomy from Denmark after a referendum in 2008. But none of those newly independent countries wanted to join the European Union. Without any explicit mention of an internal secession in the European treaties or relevant episode, we have to seek the closest mention to that situation. That is probably the article 4.2 from the Maastricht treaty as modified by the Lisbon treaty, which reads as follows:

“The Union shall respect the equality of Member States before the Treaties as well as their national identities, inherent in their fundamental structures, political and constitutional, inclusive of regional and local self-government. It shall respect their essential State functions, including ensuring the territorial integrity of the State, maintaining law and order and safeguarding national security. In particular, national security remains the sole responsibility of each Member State.”^6^

We may also add a mention to a custom in the international law: leaving a state which is a member of an international organization implies to leave the organization to which it belongs as well. The European Commission did not formally indicate what conduct the EU would adopt should a European region become independent from a member state. However, Romano Prodi, as the president of the European Commission, declared in 2004 that a region leaving a member state would leave simultaneously the European Union. His successors José Manuel Barroso and Jean-Claude Juncker confirmed that statement in several occasions.

It implies that a new state would need the unanimous agreement of the EU member states to join the organization. Now, should Catalonia or Scotland become independent states and applying to the European Union, it is unclear whether they would then follow a fast or a long track process to join the EU. What is clear is that the uncertainty does not deter pro-independence leaders from publicly considering the EU membership as self-evident.^7^

However, none of the major pro-independence political forces in Scotland and Catalonia has promised an immediate separation. The program for an independent Scotland redacted on 18 September 2014, and referendum had set the independence in March 2016 as a result of a negotiation with the central state, that is 18 months after the vote. Similarly, the Catalan pro-independence coalition Junts pel Sí (together for the Yes) of the new president Carles Puigdemont also includes a proclamation of sovereignty within 18 months after his election, which took place on 10 January 2016.

Two main conditions seem necessary to favor an international recognition. The first one is a negotiation with the central State: It would certainly have taken place for Scotland should the “Yes” have won, whereas no negotiation ever took place between Catalonia and the central government. The second condition is a ratification of the declaration of independence through a binding referendum. Catalonia has never organized any binding referendum on its independence.^8^ The 1 October 2017 vote was prohibited by the Spanish justice and not recognized by the international community. It came after a succession of official demands from its Parliament to the Spanish Congress for a binding referendum in 2001, 2004, 2012, and 2013 and again on 17 January and 18 September 2014.^9^ Quite predictably, since the Spanish constitution does not allow such a referendum and a strong majority of the Spanish MPs rejected it, the Spanish Congress turned down those seven demands.

Box 1 Brexit and the threat of Scottish independence

**Table:** 

Despite their defeat in the 2014 binding independence referendum, the supporters of an independent Scotland declared to be pleased to get a surprisingly high 44.7% share of the votes, especially within the context of a high turnout of 84.6% of registered voters. Quite logically, they did consider the possibility of a new Scottish referendum and were comforted by a further electoral success of the Scottish National Party (SNP) in the British general elections of 2015. A new vote on a Scottish independence seems more likely after the “Leave” option gained in the referendum on Brexit last June 23, 2016. Indeed, David Cameron himself warned in an interview given on June 7, 2016, that the question of a Scottish referendum would be asked should the “Remain” option be clearly validated in Scotland and rejected at state level. That condition is met: after the SNP did campaign against the Brexit, the “Remain” option won with 62% of the vote in Scotland, whereas it lost with 48.1% of votes in the UK. That difference illustrates a stronger feeling of belonging to the EU in Scotland than in the rest of the UK. The day after the Brexit referendum, Scotland’s First Minister Nicola Sturgeon declared in a press conference that its result “created a significant change” since September 18, 2014, adding that the option of a new referendum on independence “was on the table”, since “it would be democratically unacceptable” to see Scotland being taken out of the EU against our will. Some scholars have argued that Scotland might take on the EU membership of UK while the rest of UK would leave the EU. Others have suggested to take the Kingdom of Denmark as an example (Hartmann 2017). Indeed, Denmark is part of the EU while the Faroe Islands and Greenland are not. Hartmann argues that, at least from a legal viewpoint, Scotland could establish a relationship with the EU after Brexit similar to the special relation between the Faroes and the EU.	

Since the international law cannot make it compulsory for a state to recognize another one and since the EU law does not explicitly refer to internal secession, the political decisions of parent states (Spain, UK, or the EU as a whole in the case of Brexit) shall be critical in shaping independence outcomes. Genuine independence requires a negotiation with the central state, which is never obvious. Besides, obtaining a wide international recognition is not a mere formality for a new state, even though the European identity of its citizens is beyond any doubt, as it is the case for Scottish and Catalan people.

We shall conclude by underlining that the decisions made by the EU are often guided by pragmatism so that one might expect a secessionist region (which would become a new state) to be granted the possibility of enjoying a “fast track” to join again the EU. But it requires good will from its member states.

## The main drivers of breakouts of nations: an insight into the economic theory of secessions

The economic theory of secession considers that both the size and the borders of nations result from the interplay of centripetal and centrifugal forces. The former are mainly due to the benefits associated to a large population size, while the latter are mainly related to the fact that large countries are likely to be more heterogeneous.^10^ In this respect, both the optimal size of a country and the optimal number of countries across the world can be defined as a trade-off between benefits of size and the costs of heterogeneity. Optimal size must be understood as the size that maximizes the average welfare of individuals (Alesina and Spolaore, 2003, p. 11). Of course, nothing ensures that existing political institutions lead to this optimal size. Besides, whether countries are ruled by democratically elected politicians or by rent-maximizing dictators (*Leviathans*) matters to explain how borders are set or redrawn. Put differently, autocratic and democratic regimes are likely to lead to different outcomes regarding the countries’ sizes as the objective of the rulers is not the same in each.

This part is organized as follows. We study in the “‘Internal exit’ and the size of nations when governments are non-democratic” section the decision to secede in a world composed of autocratic (dictatorial) governments. Then, we move in the “Optimal size of countries in a democratic setting” section to a world where border redrawing is determined “democratically.” Some extensions are presented in the “Some extensions aiming at making theory closer to the ‘real world’” section to assess the robustness of the main results presented in the “Optimal size of countries in a democratic setting” section. Finally, we discuss in the “Fiscal decentralization, conflict, and secession” section the links between decentralization and secession as the former (together with regional transfers in favor of border regions) may be a way to compensate individuals who live far from the center in peripheral regions.

### “Internal exit” and the size of nations when governments are non-democratic

Buchanan and Faith (1987) formalized secession as an “internal exit” from a given part of the population. Secession is an alternative to migration and the “voting with one’s feet” mechanism à la Tiebout (1956). The government is represented as an elite or a sharing coalition empowered to extract revenue from taxing the rest of the population and provides “order” which is seen as a “nonexclusive, lumpy, and costly good.” This public good is financed through a proportional tax on income. The ability to secede corresponds to a participatory constraint of the governed (exploited). It establishes a limit on the government’s power to tax. The tax policy can become “accommodating” under a credible threat of secession. In Buchanan and Faith (1987), the trade-off determining potential secession is based on the scope of economies of scale in public good provision, which favors centralization, and the capability of expropriation from the government, which motivates secession. However, at the equilibrium, secession never occurs because the threat of secession modifies the behavior of the sharing coalition, which makes ex post secession unnecessary.^11^ The main result is that the central government sets its tax rate so as to make the exploited just slightly better off by remaining in the federation rather than by separating (the exploited earns no fiscal surplus).^12^

Following Buchanan and Faith’s approach, Berkowitz (1997) analyzes the impact of a peripheral region’s threat of secession in a centralized federation. The federal government sets a tax rate while the peripheral region may decide to secede. The originality of the model is that each region can pay a higher tax contribution to the federal government in order to allow the latter to provide more public goods. Such a behavior has been observed in Czechoslovakia and in the USSR. This aims of course at maintaining the unity of the federation and increasing the cost of secession for the peripheral regions. The author confirms the finding of Buchanan and Faith but only if the “exit option is sufficiently high.” Otherwise, and contrary to Buchanan and Faith, it may also be that the federal government “over-pays” in order to keep the periphery in the federation. The welfare of the peripheral region (which earns a fiscal surplus) is then higher in the federation than under separation.

Alesina and Spolaore (2003, p. 69 and following) provide an interesting and intuitive discussion about the optimal number of countries when governments behave as Leviathans. Their starting point is that an unconstrained Leviathan would maximize its rents by extracting resources from as large as possible a jurisdiction (which at the limit might be the entire world). Obviously, this is not a reasonable assumption since controlling a very large territory (an empire to use their terminology) also involves costs. Indeed, the inhabitants of the regions situated at the boundaries of the empire are likely to have distinct preferences that the central government must accommodate. Besides, even dictatorial regimes must take care of the well-being part of their population to avoid facing insurrection. It is straightforward to note that if the Leviathan governments can survive with the support of less than half of their population (which is generally the case), then the size of the countries will be larger and the number of countries will be lower in autocratic than in democratic settings (see below). Alesina and Spolaore (2003, p, 72) conclude that “dictatorships are associated with inefficiently large countries, while democratization leads to break-ups of empires and secessions”.

### Optimal size of countries in a democratic setting

While Buchanan and Faith (1987) consider the government (i.e., the sharing coalition) as given—corresponding to an oligarchy or a dictatorship setting—several authors following the seminal papers by Alesina and Spolaore (1997) and Bolton and Roland (1997) suppose that both political borders and economic policy outcomes are determined through majority voting. The previous trade-off, which determines the willingness to secede, is then slightly modified. Basically, the centripetal forces, which favor centralization, remain the scale economies in the public good provision and the size of the national market which determines the private income. In contrast, the main centrifugal force, which pushes toward secession, is once again the heterogeneity of the population. However, in this literature, individuals, who are considered immobile, differ over two dimensions: Their respective income and their preference for public goods.

Alesina and Spolaore (1997)^13^ represent secession using a spatial differentiation model à la Hotelling, which induces some heterogeneity in individual preferences for the public good. The world consists in a line segment [0,1] along which inhabitants are distributed uniformly. Countries correspond to a continuous subset of this segment. Political borders and country sizes are endogenously determined. In each country, a public good is provided in fixed quantity. Its location is determined through a majority vote. Applying the median voter theorem, and given the assumption of a uniform distribution of individuals,^14^ Alesina and Spolaore (1997) show that the public good will be provided at the center of the segment representing each country. Individuals have to bear a transportation cost to enjoy public consumption. This cost increases with the distance between their position (location) in the segment and the center. Such a cost may also be viewed as a disutility endured by individuals when they are located far away from the public good. The further the inhabitants are from the center, the lower they value consumption of the public good. At the equilibrium, inhabitants at the borders are indifferent between belonging to either one of the two adjacent countries. Secessions is associated with political fragmentation, which reduces the average distance between inhabitants and the location of the public good. In other words, increasing the number of countries reduces the disutility borne from population heterogeneity. However, secessions involve a cost, which derives from the existence of economies of scale in the public good provision. As countries become smaller, the provision of public goods become costlier, but their relative quality improves, as in average the distance to the public good decreases.

The authors conclude that at the “democratic equilibrium,” allowing secessions lead to an excessive political fragmentation: The number of countries and their respective sizes, which are strictly equal given that individuals are uniformly distributed, do not correspond to the optimal solution maximizing the world’s welfare. The number of countries in the absence of side payments is larger and the size of countries smaller at the democratic equilibrium than the optimal ones. The intuition is the following: Individuals who are far away from the government pay as much as everyone else, but they do not enjoy the benefit of the public good as much as those individuals who are located close to the government. Hence, their utility is lower than that of closer individuals. Put differently, as underlined by Alesina and Spolaore (2003, p. 44), “the voting equilibrium *does* not maximize average utility but it does maximize the utility of the individual living at the border”.^15^

Bolton and Roland (1997) propose a “positive” view of the trade-off determining secession, which lays emphasis on income inequalities across regions rather than on differences in public good preferences. The centripetal force is private income, which is higher if countries are large,^16^ while the centrifugal force is population’s heterogeneity. The model features two regions and population heterogeneity is captured through the distribution of income in the two regions. The authors consider exogenous borders at the regional level. In each region, the individual income is distributed along a general^17^ continuous law. The aim of taxation is purely redistributive: A proportional income tax is raised at a rate decided through majority voting and the publicly provided private good boils down to an equal lump-sum transfer across individuals. Secession modifies the tax rate and lump sum transfer since the identity—the income level—of the median voter changes.

In Bolton and Roland (1997), secession results from the tension between political proximity and economic efficiency (see Table 1). Secession improves on average the match between public policy and individual preferences for redistribution at the regional level. However, secession is costly as it reduces private income. Bolton and Roland’s (1997) approach allows one to consider not only individual heterogeneity through the distribution of income but also regional disparities. Both the richer and the poorer regions may want to secede for of course opposite reasons: The majority in the richer region may support secession in order to pay less taxes and have less redistribution while the majority in the poorer region may support secession to implement higher tax rates and redistribution. As in Buchanan and Faith (1987), Bolton and Roland (1997) study the conditions for secession-proof fiscal policy. Their main result is that “when the preferences of political majorities across regions differ substantially over the content of these policies, break-up may be inevitable, even if it leads to efficiency losses because of the political benefits of break-up to local majorities” (Bolton and Roland 1997, p. 1084).

**Table 1:** A simple model of secession

We consider a sequential game. In the first stage, the inhabitants of a region vote about the status of their region (independence versus status quo)^a^. In the second stage, they vote about the policy to adopt at the local or central level^b^. The individuals have either different incomes or preferences about the public good. The first type of heterogeneity implies redistributive issues. The public good comes down to a lump-sum transfer, which can lead to redistributive conflicts between the individuals of a country. In this case, secession aims at reducing the level of regional inequalities (Bolton and Roland 1997). When the preferences over the public good are heterogeneous, secession modifies the quality of the public good (in terms of geographical location, individual preferences, etc.). By making the population more homogenous, it increases the average satisfaction of the voters-tax payers (Alesina and Spolaore 1997). Depending on the nature of the heterogeneity, the secession’s effect is quantitative or qualitative. However, these effects are not mutually exclusive. They can even be combined (see for instance Bolton and Roland 1996). Individuals consume both public goods and private goods, which are supposed to be perfect substitutes^c^. Moreover, the agents’ utilities are assumed to be additively separable. By nature, the public good is inevitable: No individual can avoid consuming or financing it. It can take the form of public services or government transfers. Individuals vote on a unique tax rate. Let *V*_*i*_(·) be individual *i*’s utility: $$ {\displaystyle \begin{array}{l}{V}_i:\kern0.5em {\mathrm{\mathbb{R}}}^{+}\times {\mathrm{\mathbb{R}}}^{+}\times I\mapsto \mathrm{\mathbb{R}}\\ {}\left({c}_i,g,q\right)\mapsto {c}_i+{v}_i\left(g,q\right).\end{array}} $$ *c*_*i*_ is individual *i*’s private consumption, which is equal to available income. It varies with *i*’s initial wealth (*w*_*i*_) and the constant tax rate (*t*). We have *c*_*i*_ = (1 − *t*)*w*_*i*_. Individual income (*w*_*i*_) is initially exogenous and certain, and subsequently becomes random or even endogenous. *g* is the quantity of public good provided in the jurisdiction. It can be constant or endogenous, in which case it varies with the tax rate (*t*) and the tax base (*Y*)^d^: *g* = *g*(*t*, *Y*). We keep the general expression *g*. *q*, an *n*-tuple of (*I*)^e^, represents the quality of the public good. Following Hotelling and Lancaster, we assume that each individual has an ideal level of public good. The function *v*_*i*_(*g*, *q*) of *ℝ*^+^ × *I* in *ℝ*represents the satisfaction of the individual *i*, consuming a quantity *g* of the public good of quality *q*. All the authors cited assume implicitly that a change in the quality (*q*) does not change the cost of provision. The endogenous variable (s) (*t*_*m*_, *g*_*m*_, *or q*_*m*_) are chosen by majority vote. They correspond to the median preferences of the population^f^. Individual *i*’s indirect utility, where the price *t* and the quantity *g* of the public good are taken into account, can be written as *U*_*i*_(*t*_*m*_, *g*_*m*_, *q*_*m*_; *w*_*i*_) = (1 − *t*_*m*_) · *w*_*i*_ + *v*_*i*_(*g*_*m*_, *q*_*m*_) We put an index *s* on the different variables after secession. Secession modifies the quantity and the quality of the public good. It also affects the individual income: ${w}_i^s\equiv \alpha \left({w}_i\right)$, with 0<*α*(*w*_*i*_) ≤ *w*_*i*_. Individual *i*’s utility becomes ${U}_i^s\left({t}_m^s,{g}_m^s,{q}_m^s;\alpha \left({w}_i\right)\right)=\left(1-{t}_m^s\right)\cdotp \alpha \left({w}_i\right)+{v}_i\left({g}_m^s,{q}_m^s\right)$ where ${t}_m^s$ and ${q}_m^s$ are determined by the vote of the secessionist population. Individual *i* wants independence iff it provides him a higher utility level. The utility difference can be written as $$ {\displaystyle \begin{array}{l}\Delta \kern0.5em {U}_i\equiv \kern0.5em {U}_i^s\left({t}_m^s,{g}_m^s,{q}_m^s;\alpha \left({w}_i\right)\right)-{U}_i\left({t}_m,{g}_m,{q}_m;{w}_i\right);\\ {}\Delta\ {U}_i=\underset{E_i^r}{\underbrace{\left(1-{t}_m^s\right)\cdot \left(\alpha \left({w}_i\right)-{w}_i\right)}}+\underset{E_i^f}{\underbrace{\left({t}_m-{t}_m^s\right){w}_i+{v}_i\left({g}_m^s,{q}_m\right)-{v}_i\left({g}_m,{q}_m\right)}}\\ {}+\underset{E_i^p}{\underbrace{v_i\left({g}_m^s,{q}_m^s\right)-{v}_i\left({g}_m^s,{q}_m\right)}}\end{array}} $$ Independence has three effects: an income effect (${E}_i^r$), a fiscal effect (${E}_i^f$), and a political effect (${E}_i^p\Big).$ The first term, ${E}_i^r$, is the individual income loss. It is an immediate consequence of the hypothesis of a secession cost. We conclude that ${E}_i^r\le 0$ from *w*_*i*_ ≥ *α*(*w*_*i*_). The tax effect, ${E}_i^f$, is a quantitative one. It can be decomposed into two terms. $\left({t}_m-{t}_m^s\right){w}_i$evaluates the variation of tax rates. Its sign depends on the presence of potential economies of scale in the production of the public good and the preferences of the new median voter. In general, the decrease in the size of the country, and hence the tax base, implies an increase in the tax rate, i.e., ${t}_m^s>{t}_m$. The second element, ${v}_i\left({g}_m^s,{q}_m\right)-{v}_i\left({g}_m,{q}_m\right)$, measures the variation of the quantity of the public good, given constant quality. Its sign depends on the joint evaluation of the tax base (*Y*) and the preferred tax rate (${t}_m^s$ and *t*_*m*_). The political effect, ${E}_i^p$, is qualitative. It results from the change in the identity of the political decision-maker in equilibrium. If the effect is positive, secession (or decentralization) makes policy-makers choose a public policy that is closer to the ideal policy of agent *i*. The following table summarizes the contributions of the two most important papers: Alesina and Spolaore (1997) and Bolton and Roland (1997). For Alesina and Spolaore (1997), political borders are determined by a trade-off between the tax effect and the political effect. For Bolton and Roland (1997), the trade-off is between the income effect and the fiscal effect. For the former, secession has a purely allocative goal by moving the local public goods closer to the individuals that finances it. In contrast, the latter emphasize the redistributive effect resulting from a political separation.		
	Alesina and Spolaore (1997)	Bolton and Roland (1997)
Political borders	endogenous	exogenous
Nature of heterogeneity	assessment of public good	income
${E}_i^r$	0	(*α* − 1)*w*_*i*_ < 0
${E}_i^f$	(*t* − *t*^*s*^)*w* < 0	$\left(t-{\alpha}_t^s\right){w}_i+{g}^s-g\gtrless 0$
${E}_i^p$	a.g.$\left({q}_i-{q}_i^s\right)\gtrless 0$	0

^a^This simple model is drawn and translated from Rota-Graziosi (2004)

^b^This can require several votes

^c^The complementarity of private and public goods is not explicitly covered in this literature review (c. f. Casella (1994 and 2001); Casella and Feinstein (2002))

^d^With $Y={\int}_{\underset{\_}{w}}^{\overline{w}}{w}_ih\left({w}_i\right)d{w}_i$, where *h*(·) is the income distribution in the jurisdiction on the support $\underset{\_}{\Big[w},\overline{w}\Big]$

^e^*q* = (*q*_1_, *q*_2_, …, *q*_*n*_), where *q*_*i*_ represents the characteristic *i* of the public good. (*I*) is a set of dimension *n* that gathers all the possible values of the *n* characteristics of the public good. Given the conditions on the application of the median voter theorem, the set (*I*) is often reduced to a single dimension, for example the geographic location

^f^If the preference space is multidimensional, it is usually difficult to determine the median quality. We assume that each characteristic of the public good is decided upon by vote. However, given this assumption, the results vary depending on the agenda of the votes. The models considered in this literature review do not exceed two-dimensionality

### Some extensions aiming at making theory closer to the “real world”

The models presented above ignored (or at least left aside) major questions that are nevertheless relevant in a more “positive” perspective of the secessionist phenomena and that we analyze in the following.

Globalization is intuitively a matter of interest for the study of secessions. The relationship between economic integration and political fragmentation has been largely studied (see Casella 1994, 2001; Alesina and Spolaore, 1997; Alesina et al. 2000). Economic integration mitigates an advantage attributed to large countries, namely their large market size. Indeed, in a globalized world, the size of political jurisdictions does not need to coincide with the size of their market as trade is free across countries. Hence, economic integration across countries intended to reduce trade costs should also favor secession in these countries as the market potential of small-sized countries would be larger than their domestic size.^18^ It would be straightforward to show formally that both the optimal number of countries (which maximizes the average utility) and the number of countries at democratic equilibrium are increasing with trade openness (note that the number of countries in a Leviathan equilibrium also rises with trade openness). One may go further and argue that the relationship between “smallness” and trade openness is reinforcing each other as small countries need being more integrated in the world markets.

It has been assumed so far that public goods were not subject to interjurisdictional spill-overs, which seems to be an “heroic” assumption. Ellingsen (1998) deals with the impact of interjurisdictional externalities and free riding on the decision for two regions to keep full sovereignty or alternatively form a union. Unification takes place if there is a pro-union majority in each region. In each jurisdiction, the government provides a non-excludable public good that maximizes the utility of the majority. The “small” jurisdiction may free ride some non-excludable public goods provided by the large one. It is well known that the benefits of public goods provided by large and central jurisdictions may spill over into neighboring smaller ones. The authors show that the small region tends to oppose unification with the larger one and this is robust to different specifications of tastes for the public good. The corollary of this result is that free riding from larger neighbors is an additional factor pushing small regions to secede.

The introduction of strategic voting behavior in the economic theory of secession also leads us to consider strategic delegation by the median voter toward a representative who has not the same preferences as him (her) in terms of public spending.^19^ In this line, Gradstein (2004) considers the secession option in a Coasian bargaining between two regions that are members of a federation. The author highlights that secession or union decision involves a strategic delegation issue. Through strategic delegation, the median voter of each region tries to reduce her own contribution in order to push the others to support a larger share of the tax burden to finance the federal public good. This behavior leads to an inefficient allocation of resources in the federation, which may be “unattractive” for the minority region. Indeed, in each region, the electorate chooses a representative who has a lower preference for public goods than the general population. Such a move is a commitment (à la Schelling), which aims to improve regions’ bargaining power in determining the package of tax rate and public good provision. Limiting the secessionist tendencies by imposing a referendum at the regional level instead of allowing the representative to decide alone would restore efficiency.

Finally, risk and information asymmetry can be introduced in the economic analysis of secession. Typically, political union allows to some extent risk pooling, which reduces individuals’ income uncertainty. However, risk pooling may also generate a well-known moral hazard problem since regions may have less incentives to implement policies that decrease national risk. Van Hagen and Eichengreen (1996) analyze such a trade-off between risk pooling and moral hazard behavior in the case of the European Union. Another trade-off related to uncertainty is the one studied by Persson and Tabellini (1996). The latter consider two kinds of risk that each individual is facing: An economic risk and a political risk. The former is a centripetal force since some insurance (risk pooling) is possible across the regions of the same country. The latter derives from the diversity of voters in a large union. This leads to uncertainty about the median voter’s income and then the public policy that is eventually chosen. Political uncertainty increases with the size of the country and constitutes the main centrifugal (secessionist) force. Secession mitigates political uncertainty in reducing discrepancies between voters. However, it comes at the expense of the inter-regional insurance mechanism.

### Fiscal decentralization, conflict, and secession

Secession can be seen as an extreme case of decentralization, when all the prerogatives of the State (tax and spending) are transferred to the local political unit. The literature on fiscal federalism has largely debated the (in) efficiency of decentralization (see Martinez-Vazquez et al. (2016) for a survey). All the previous mechanisms determining the occurrence of secession are also at play when it comes to define the “optimal” architecture of the state. On the spending side, some public goods may be locally delivered to better match populations’ preferences while others should remain supplied centrally to avoid duplication costs or the loss of scale economies (Oates 1972). On the revenue side, granting subnational governments with more autonomy is expected to improve the accountability of local officials, but it would also induce a risk of harmful tax competition named “race to the bottom” (see the seminal paper by Zodrow and Mieszkovski 1986).

Panizza (1999) develops a model where individuals consume a private good and two public goods: A national public good (defense) which is subject to economies of scale (its average cost is decreasing with the country’s size) and a local public good (education). Education is provided by both the central government and subnational units. Individuals prefer local public goods that are provided by subnational jurisdictions (as it fits their preferences best) to public goods provided by the central government. Decisions over the public goods are made according to the preferences of the median voter in the respective jurisdictions. All things being equal, the more decentralized the country is (i.e., the higher the share of education that is locally provided), the higher is the utility of individuals. However, subnational jurisdictions are less efficient at providing the national public good (defense), and there is thus a trade-off between quality of match between preferences and the provision of the local public good (highest in decentralization) and the cost of providing the national public good (also highest in decentralization). Subnational jurisdictions will decide to secede if the utility enjoyed by the regional median voter by locally providing the local public good (education) outweighs the disutility resulting from a higher cost of providing the national public good (whose financing is not shared any longer with the other jurisdictions in case of secession).^20^

Provided that the central government wants to avoid secession by all means, it will choose an equilibrium level of decentralization which conveys the same utility to individuals that they would enjoy with secession. Then, decentralization substitutes for separation, and unsurprisingly, the more heterogeneous the country is with respect to tastes for education, the more decentralized it will be. Arzaghi and Henderson (2005) obtain the same kind of results using a two regions model where the central region never claims for separation (greater autonomy) while the peripheral region may do so as its population endures a loss of utility in a unitary structure due to spatial decay. Spatial decay refers to the fact that public good supplied by a central government may not fully reach distant minorities because of the difficulty to monitor the delivery of the public good over long distance and of the inadequacy of one-size-fits-all provision in the face of local specificities. Then, the level of local public good available to individuals in the peripheral region turns out to be lower than in the central region.

In the case of a unitary country, the government is assumed to be located in the center where the median voter also is. There is no real secession in this model as separation means that the country shifts from a unitary structure (where the central government sets the same tax rate and the same level of provision of local public good across the country) to a federal arrangement. Spatial decay vanishes in a federal system since each region has to provide and finance the local public good for its own constituents only. On the other hand, the demand for greater autonomy will also depend on the cost of running a new government for the peripheral region. Spolaore (2010) also argues that decentralization reduces the cost of heterogeneity and then reduces incentives to secede (centripetal effect). However, decentralization has also a centrifugal effect when it allows the secessionist regions to get access to additional resources at the expense of the central government (the odd of success of separation depends on the conflict capabilities of the central government and the peripheral region). Spolaore (2010) shows that if the country is highly centralized (above a given threshold which depends on the parameters of the model), then decentralization is likely to foster separation while a decentralized country will take advantage of decentralizing more to prevent secession.^21^,^22^

## Empirical evidence about the determinants of secessions and the means to deter border redrawing

In the first part of this section, we aim to review the empirical determinants of secessions and compare these to theoretical predictions. In the second part, we will explore whether federalism and decentralization are likely to deter separatist claims when countries are very ethnically heterogeneous.

### The main determinants of separation claims

The theoretical literature suggests that inhabitants of a given region will prefer staying part of a larger country (or a union) when the associated net benefits of the status quo outweigh the net benefits of separation. The theoretical discussion in the “The main drivers of breakouts of nations: an insight into the economic theory of secessions” section highlighted a key trade-off between two forces: increasing returns to scale in the provision of public goods, on the one hand, and cultural and preference heterogeneity across individuals living in different regions, on the other hand. The former force discourages regions to secede, to take advantage of the economic benefits induced by large country size.^23^ The latter force encourages regional breakup as it is difficult for large and heterogeneous countries to have their policies match with the preferences of everyone, especially regional groups.

It is worth noting that the aforementioned trade-off is similar to the one put forward by the fiscal federalism literature to define the optimal degree of fiscal decentralization (Oates 1972). In both cases, there is a trade-off between the presence of economies of scale in the provision of public goods and heterogeneity of preferences. Panizza (1999) argues that the devolution of power by central governments to subnational governments is a way to preserve territorial integrity of the country when secessionist claims are credible. Arzaghi and Henderson (2005) explicitly model regional demands for decentralization drawing upon the literature on secessions. Separation in their model not only means a shift from a “strong unitary system to a strong federal system” but also leads to a situation where regional governments have full discretion to provide public goods and no public goods are provided by the central government any longer, which eventually comes down to separation.

Drawing from both the literature on secession and fiscal decentralization leads us to focus on three core assumptions: H1: Large regions will display more secessionist tendencies than smaller ones*.* This is because large regions would form a country that can still benefit from scale economies in the provision of public goods. H2a: Regions richer than the average of the existing country will be more likely to exhibit secessionist tendencies than poorer regions. This is because richer regions usually are net contributors to fiscal equalization schemes. Richer regions would also form independent countries that are more viable economically. H2b: Regions will display more secessionist tendencies as the country becomes richer*.* There are fixed costs involved with the process of separation (e.g., setting-up an administration), so higher income makes these costs affordable. H3a: Regions which are culturally distant from the rest of the country will display more secessionist tendencies. This is because their preferences may be quite far from those of other regions and groups. Thus, policies tailored for the median voter in the country may not differ from minority groups’ preferences. H3b: Countries with large and diverse populations will be more prone to secessionist demands than smaller, more homogenous countries. This is because it is more challenging for a central government to accommodate heterogeneity of preferences across the territory.

A first set of papers does not explicitly deal with the determinants of secession but rather aims at explaining the optimal level of centralization (resp. decentralization). As argued above, these results are of interest to help us understand why some regions decide to separate. Panizza (1999) conducts a cross-sectional analysis on a sample of 55 countries and finds that the central government’s share of revenues (used as a measure of fiscal centralization) significantly decreases with the size of countries, per capita income levels, and their degree of ethnic fragmentation (although the latter effect is less robust). Using similar data, Arzaghi and Henderson (2005) find that federalism is more likely in rich and large countries. They also find that fiscal decentralization is positively associated with the size of countries, both in terms of population and area. However, no association can be drawn from the data between ethnic heterogeneity and decentralization. Sambanis and Milanovic (2014) conduct a similar study on self-determination movements in 48 decentralized countries and 876 regions over the period 1945–2012. They explicitly assume that regions that enjoy a status of autonomy must have expressed secessionist will (as central governments are unlikely to grant territorial autonomy otherwise). The share of regional public expenditures that is financed from the regions’ own resources is used a proxy of autonomy.^24^ They confirm that richer and more populous regions are more likely to express sovereignty demands than poorer and smaller regions. Moreover, regions with natural resources (oil and minerals) are also more likely to be autonomous.^25^ However, they do not find an obvious relationship between the ethnic distinctiveness of regions and their likelihood of being autonomous.

Wimmer et al. (2009) use the Ethnic Power Relations (EPR) dataset to explain secessionist claims of ethnic groups in all countries of the world from World War II to 2005. They show that secessionist wars are more likely to break out for those ethnic groups which are excluded from participation in central state power, which had an imperial past and a long-standing history of indirect rule (a large part of the population is not used to be governed and to report to the political center), and which live in large countries with high levels of linguistic fractionalization. Wimmer et al. (2009) is not informative on the determinants of secessionist demands themselves. Whether secessionist demands lead to civil war or not may itself depend upon the same range of factors. Cunningham (2013) directly investigates why some self-determination movements turn violent, whereas some others lead to non-violent protest and others yet remain within the confines of conventional politics. Interestingly, she finds that violence is more likely to occur over conventional politics when the groups that demand self-determination are large, have kin in adjacent states, are excluded from political power, are economically discriminated, and are relatively poor. Apart from this last variable, these results are consistent with Wimmer et al.’s (2009), and so the findings on secessionist conflicts can be considered informative on underlying demands for separation.^26^ This is further confirmed by Sorens (2005) who focuses on non-violent avenues to separation. He shows that the vote share of secessionist parties in advanced democracies increases in regions where a distinct language is dominant, which are geographically removed from the political center, with a history of independence, and which concentrate a high share of the country’s population and GDP. Empirical findings appear to be largely consistent and in line with theoretical predictions.

Another strand of the literature aims at estimating the trade-off between economies of scale and cultural heterogeneity. This offers a direct test of theoretical predictions. Desmet et al. (2011) estimate such a trade-off by calibrating a structural model that predicts the likelihood that a region prefers to remain part of a country (or, alternatively, prefers independence). By the same token, the model allows the researchers to study under which conditions countries may cooperate with each other or even unite. In the model, the world is composed of countries, which themselves are divided into regions in which agents live. The latter are immobile and have different incomes. They vote on the optimal level of a public good which is produced under increasing returns to scale. Utility derived from the consumption of the public goods is supposed to decrease with the cultural heterogeneity of the country (homogeneity is supposed within regions). The tax rate in each country is chosen by majority voting. Secession may occur unilaterally or require the majority of each region affected by the separation process. The model is calibrated by assuming that the current European map is stable. The key variables of the model are economic development (GDP per capita), population size, and a parameter summing up the cost of cultural heterogeneity (proxied by genetic heterogeneity).

Their results are the following: (i) The Basque country and Scotland are the regions that are the most likely to secede (which interestingly is consistent with the real situation of these two regions); (ii) unsurprisingly, countries which are quite similar in terms of culture, population, and GDP are the most likely to unite. This would be more specifically the case of Switzerland and Austria, Denmark and Norway, or Belgium and Netherlands; (iii) separatist claims can be contained even within highly unequal unions as long as cultural differences are either limited or are not negatively valued by the population; and (iv) ex-Yugoslavia is a good laboratory to test for some predictions of the model. Based on pre-war levels of GDP per capita and cultural distance in the ex-Yugoslavia, the authors observe that for reasonable values of the cost of heterogeneity, Slovenia would have had a small gain of leaving the union. This was due to Slovenia being by far the richest country of the Yugoslav republics (at that time, it was twice as rich as the second richest one, i.e., Croatia). Once they allow Slovenia to leave the union, their model now predicts that Croatia too has an interest in seceding. This is logical as the union minus Slovenia brings less benefits to the other constituents and Croatia would now be the richest republic (it also was twice as rich as the remaining four constituencies). After both Slovenia and Croatia leave, the model predicts that the process of disintegration can either stop (if the cost of heterogeneity is low) or continue further (if the cost of heterogeneity is high). In the latter case, Bosnia and then Macedonia would eventually secede, leaving Yugoslavia restricted to its republics of Serbia and Montenegro.

This fits quite well with the historical events. Indeed, Bosnia, Croatia, Slovenia, and Macedonia have all seceded in 1991 or 1992. Montenegro, which is the least likely to quit the union in the model, only declared independence in 2006. Interestingly, the calibration of Desmet et al. (2011) found that the complete breakup of Yugoslavia was a relatively likely event. Of all the countries and regions of Europe, the most likely secessions were to be found in the ex-Yugoslavia. This was largely due to the large economic differences that existed in this union. Cultural differences, in contrast, were quite modest (on average genetic distances were no higher than, say, between England and Scotland) so that the key for the process of disintegration to unfold was that these differences mattered a lot to the people (in a negative way).

It is interesting to conclude this section to get insight into the economic impact of independence. Reynaerts and Vanschoonbeek (2016) compare the economic growth trajectory of newly independent countries to the growth trajectory of a synthetic control group representing what would have happened in the post-independence period, had the secession not occurred. They show that independence generates long-term economic costs as the newly independent countries tend to experience a lower rate of economic growth than they would have experienced had they remained in the union. As a result, GDP per capita in newly independent countries is 20% lower, on average, than what it would have been without separation. This is consistent with the view that smaller constituencies are less efficient economically. For a region, breaking-up from large polities carries an economic cost which individuals may nevertheless be willing to bear if they value cultural heterogeneity very negatively. Reynaerts and Vanschoonbeek (2016) also show that the cost of independence increases with the size of the country (likely due to reduction in scale economies) but that this cost can be partly mitigated by heightened openness to trade of the new entity.

### Decentralization: a means to accommodate ethnic diversity and deter secessionist conflicts?

Most central governments dislike granting autonomy, let alone independence, to specific ethnic groups for fear of contagion to other groups and loss of territorial integrity (Walter 2006). Demands for sovereignty are thus commonly met with resistance, which can lead to violence. For instance, Sambanis and Milanovic (2014) found that out of 221 regions enjoying some degree of autonomy in their sample, 46 experienced violent relations with the central government. There is a strong presumption that conflicts over territories are difficult to solve peacefully as it is not always practical to divide territories across actors. Disputes are especially intractable when territories are imbued with symbolic or strategic values, such as access to sea, mining locations, or military bases (Toft, 2003). Fearon and Laitin (1999) find, for instance, that territorially concentrated minorities are far more likely to experience large-scale violence than other groups. It is therefore not surprising that about half of all reported ethnic civil wars that occurred since 1945 were secessionist wars (Wimmer et al. 2009).^27^ Conversely, Wimmer et al. (2009) found that nearly all secessionist wars in their sample were ethnic in nature (only 3 out of 60 were not). It is important to stress that ethnicity is not viewed here in the common, narrow sense of the word. Scholars since at least Horowitz (1985) use a very broad definition of ethnicity that applies to groups based on mostly hereditary traits (or “ascriptive” traits) that include language, tribe, religion, nationalities, and even castes (see Chandra 2004 for a discussion of the different definitions). The following discussion should therefore not be seen as solely confined to narrowly defined ethnic conflicts.^28^

To manage “territorial” cleavages and prevent secessions, decentralization can be an appealing solution. It allows central governments to grant some degree of autonomy and decision power to regions or ethnic groups that wish to secede, without loss of sovereignty. Many scholars have advocated decentralization for that reason (e.g., Lijphart 1977; Nordquist 1998; Hechter 2000; Bermeo 2002; Hooghe 2004; Gurr 1994). In contrast, opponents point out that decentralization in multi-ethnic countries contributes to “freeze” ethnic identities over time (Hardgrave 1993, Kymlicka 1998) and to reinforce the legitimacy of ethnically defined subunits (Cornell 2002). Moreover, decentralization provides new institutional and economic resources to the separatist movements through the regional governments. Thus, decentralization may well foster—and not prevent—violent conflicts (Cornell 2002, Roeder 1991, Bunce 1999, Snyder 2000). Ethno-federalism, which refers to a situation where regions are crafted to grant ethnic groups control of regional government, has notably been blamed for secessionist tendencies in the ex-Soviet Union and ex-Yugoslavia (e.g., Roeder 1991, Bunce 1999; Cornell 2002) and for the failure to contain ethnic violence in Nigeria (Suberu 2001). Hale (2004) argues that ethno-federal arrangements in which a specific region is dominant are destabilizing.

The empirical literature on decentralization and conflict has grown since the 1990s. A strand of this literature uses ethnic groups as the unit of analysis, mostly through the “Minorities At Risk” (MAR) and Ethno-Power Relations (EPR) databases.^29^ Cohen (1997) finds that federalism is associated with less rebellion but more protest, perhaps indicative that with decentralization, conflicts shift from the center (where stakes are high) to the local level (where stakes are lower). Likewise, the estimations of Saideman et al. (2002) suggest that federalism reduces ethnic rebellion. These studies suffer, however, from endogeneity issue. As we have seen, decentralization is likely to be explained by conflict as much as the other way around. Results from simple pooled analyses should thus be taken with caution. Studies by Tranchant (2008, 2016), Christin and Hug (2012), and Cederman et al. (2015) attempt to address the endogeneity with quasi-experimental methods.

Tranchant (2008) looks at fiscal decentralization, which is captured by the share of subnational expenditures in general government spending collected by the International Monetary Fund. Results on 50 ethnic groups between 1985 and 2001 show that fiscal decentralization consistently reduces ethnic rebellion and intergroup violence. The positive (i.e., in the sense of conflict mitigating) impact of decentralization is maximized in countries with high GDP per capita.^30^ Cederman et al. (2015) and Tranchant (2016) use the Ethno-Power Relations (EPR) dataset on all 800 politically relevant ethnic groups worldwide and find that territorial autonomy and fiscal decentralization, respectively, tend to reduce ethnic civil wars. Both explicitly deal with endogeneity (through instrumental variables and difference-GMM estimators).

Another strand of the literature resorts to cross-country analyses, pooling together information on all ethnic groups within a country. This obscures critical heterogeneity (for instance, the protracted armed conflict in Aceh involved a region which makes up less than 2% of Indonesian population), but it avoids sample selection bias issues.^31^ Among country-level studies, three especially important ones are Bakke and Wibbels (2006), Brancati (2006), and Christin and Hug (2012). Bakke and Wibbels (2006) focus on 22 federal or semi-federal states between 1978 and 2000 and consider (among others) how regional inequalities and fiscal transfers across regions mediate the impact of federalism on civil wars (based on the Armed Conflict Dataset) and on ethnic rebellion and protest (both based on MAR). They find that federalism in the context of large regional inequalities contributes to ethnic rebellion and protest but that fiscal transfers to regionally concentrated ethnic groups detracts from armed conflict. Brancati (2006) focuses on 30 democratic states and investigates the direct and indirect effects of political decentralization. Direct effects are hypothesized to operate following the preference-matching mechanism described above. Indirect effects of decentralization are assumed to be negative and to operate through the strength of regional parties. The argument of Brancati (2006) is that decentralization allows regional parties to strive as it gives them opportunities to win elections and influence policy. Insofar as strong regional parties contribute to freezing and legitimating ethnic identities, they also favor secessionism and conflict. Using unique data on regional parties and MAR data on ethnic violence (aggregated at country level), Brancati (2006) shows that both direct and indirect effects are statistically significant and have the expected signs. The net effect of decentralization is then peace preserving if decentralization is not accompanied with rising strength of regional parties and conflict productive otherwise. Christin and Hug (2012) find that countries with a strong ethno-federal structure (i.e., where minorities control a large share of subfederal units) such as Nigeria or Brazil are the most prone to experience an onset of ethnic conflicts. This is also consistent with the view expressed by Horowitz (1985) that federalism along ethnic lines is destabilizing.

## Conclusion

We have seen that economists predict secessions when economies of scale are relatively unimportant and the cost of population heterogeneity is high. Small economies of scale mean that newly formed countries will not suffer much from their smaller size when providing public goods. High costs of population heterogeneity mean that newly independent countries will benefit from a greater social cohesion. Secessionist claims are also predicted to be more likely from richer-than-average regions, which subsidize the rest of the country. The patterns of secessionist demands analyzed in the empirical literature seem largely consistent with this economic reasoning. Regions that have the least to lose from breaking-up from a union, and which are most likely to succeed economically on their own, are the most likely to express secessionist tendencies. However, the desire of self-rule cannot be explained solely by a narrow economic calculus. In fact, we have seen that individuals in newly independent countries bear a large economic penalty from the secession (equivalent to about 20% of the GDP per capita level). We have also seen that economic differences alone would probably not have sufficed for the ex-Yugoslavia’s disintegration process to happen. This shows that self-rule may offer intrinsic value to individuals, especially for members of culturally distinct groups.

Legally speaking, the international law promotes two seemingly contradicting rights regarding secession: A right of self-determination and a principle of states’ territorial integrity. In some occasions, the international community considered that the first principle dominated the second and accepted separations as a legitimate outcome. This was the case of the decolonization process and for the separation of East Timor (1999), Bougainville (2001), or South Sudan (2001). A history of discriminations and free and fair binding self-determination referendums seem to be a necessary—but not sufficient—condition for the international community to recognize newly created states. Within the EU, no formal stance has been taken regarding secessions of member states or from within member states, so that outcomes of secessionist demands will critically hinge on political decisions by member states.

We have also seen that countries that are large, diverse, and regionally unequal are especially likely to resort to decentralization, largely in a bid to contain secessionist demands. Empirical studies largely agree that federal and decentralized arrangements can successfully contain centrifugal forces, under certain conditions. Federal systems in which regional borders closely coincide with the distribution of ethnic groups, for instance, are likely to be unstable, especially when one region is dominant. However, the granting of regional autonomy status and/or the devolution of substantial spending and taxation powers to subnational governments are associated with fewer ethnic conflicts, especially in democratic and rich countries. There is still a question mark as to the effectiveness and desirability of these institutional devise in poor and/or fragile countries.

## Footnotes

1 In the following lines, we will define a nation-state by three principles: sovereignty understood as the supreme power to express the general will (Rousseau 2014), independence in the management of their internal affairs, and mutual recognition. That definition implies that nation-states conserve a key role in self-determination processes.

2 The full text of 1514 Resolution can be downloaded at the URL: http://www.sfu.ca/~palys/UN-Resolution%201514.pdf.

3 United Nations Declaration on the right of indigenous peoples, http://www.un.org/esa/socdev/unpfii/documents/DRIPS_en.pdf.

4 Roethke (2011, p. 38) underlines that “some scholars argue that territorial integrity merely safeguards the inviolability of international borders but does not regulate an internal affair such as secession”.

5 Sources: Amaël Cattaruzza, “Les référendums d’autodétermination: démocratisation ou balkanisation du monde?”, *L’Espace Politique* [Online], 3 | 2007-3, posted on 22 December 2007, URL: http://espacepolitique.revues.org/914 - BBC, “Scotland decides”, page on the Scottish referendum on independence held on 18 September 2014, [Online], URL http://www.bbc.com/news/events/scotland-decides/results - Fondation Robert Schuman, Corinne Deloy, “After twenty four years of union with Serbia, Montenegro is independent again”, 22 May 2006, [Online], URL http://www.robert-schuman.eu/en/eem/0522-after-twenty-four-years-of-union-with-serbia-montenegro-is-independent-again - International Crisis Group “ Indonesia: history lessons”, 11 April 2013, [Online], URL https://www.crisisgroup.org/asia/south-east-asia/indonesia/history-lessons - Vincent Laborderie, Nicolas Parent (Dir), 2012, *Good morning Belgium*, Editions Mols, Brussels, 210 p. - Sonia Le Gouriellec, “Trois trajectoires de sécession dans la Corne de l’Afrique: le Somaliland, l’Érythrée, le Soudan du Sud”, *Sécurité globale*, 2011/4 (N° 18), p. 95-106. - Stéphane Rosière, “La fragmentation de l’espace étatique mondial”, *L’Espace Politique* [Online], 11 | 2010-2, posted on 18 November 2010, URL: http://espacepolitique.revues.org/1608 - Southern Sudan Referendum 2011, official results validated by the Southern Sudan Referendum Commission, [Online], http://southernsudan2011.com/ - David Torrance, 2013, *The Battle for Britain. Scotland and the Independence Referendum*, Biteback Publishing, London, 370 p.​

6 EU Lisbon treaty in the official EU website: http://eur-lex.europa.eu/legal-content/EN/TXT/?uri=CELEX%3A12012M%2FTXT.

7 This uncertainty has also been a strategic device to deter secession by the anti-independence leaders.

8 The Catalan regional elections of 27 September 2015 were interpreted as a referendum by the autonomous government. The elections were marked by an unprecedented turnout of 77.44%. Indeed, in Catalonia, regional elections usually have a lower turnout than general elections since a part of the registered voters feel more involved in the latter than in the former. The previous Catalan regional elections of 25 November 2012 were marked by a 67.76% turnout, which was already high. Afterwards, the unofficial and unbinding consultation on the independence of Catalonia organized on 9 November 2014 obtained 2.34 million voters while the regional elections of 27 September 2015 mobilized 4.11 million voters on a 7.5 million people population. That unprecedented mobilization could be observed on any geographical scale. We may compare the electoral mobilization in Catalonia to the binding referendum on the Scottish independence last 18 September 2014 and its turnout of 84.6%.

9 Congreso de los Diputados: http://www.congreso.es/portal/page/portal/Congreso/Congreso/Iniciativas

10 According to Alesina and Spolaore (2003, p. 3–4), benefits enjoyed by large sized countries are the following: (i) The cost of providing certain public goods is decreasing with the number of people and tax payers (this is especially true for pure public goods such as defense and security), (ii) large countries can raise more resources and better defend their frontiers, and (iii) large countries are generally richer than smaller ones because they have larger markets and may take advantage of a “home market effect” as shown by the new economic geography literature. Furthermore, the “home market effect” is one of the main drivers of the agglomeration processes; (iv) large countries often provide “insurance” to the regions that are harmed by economic downturns or natural calamities.

11 The equilibrium is said to be secession-proof. A symmetric notion of coalition-proofness Nash equilibrium has been studied in game theory by Bernheim et al. (1987), among others.

12 Rota-Graziosi (2007) emphasizes that a private income effect has also to be considered in Buchanan and Faith (1987) framework, since income is an increasing function of the size of the market (which perfectly matches the size of the jurisdiction). This private income effect favors centralization or equivalently reduces the threat of secession and increases the government’s capacity of exploitation.

13 We present these papers in the order as they appear in the same issue of the *Quarterly Journal of Economics* (see references at the end of the paper).

14 A uniform distribution is symmetric: which means that its median and its mean are identical.

15 The result of Alesina and Spolaore (1997) is however very sensitive to several assumptions, in particular the fixed cost of public goods with respect to the unitary transportation cost.

16 By assumption, secession reduces the size of the domestic market and consequently individual private income. Indeed, the authors assume some efficiency losses with secession given that “any allocation that is achieved under separation can be replicated in the unified nation by introducing the same degree of decentralization as under separation.”

17 The income distribution, which will determine the preference for public policy, is not here systematically symmetric.

18 It is noteworthy that an implicit assumption made by the seminal papers presented above is that the successor countries would enjoy the same access to the worldwide market than the initial State.

19 Such sophisticated voting behaviors were considered neither by Alesina and Spolaore (1997) nor by Bolton and Roland (1997).

20 See in the empirical part below Desmet et al. (2011) who calibrate a structural model for the EU.

21 Flamand (2015) uses mostly the same ingredients as the previous literature and develops a model à la Alesina and Spolaore (1997) where income inequalities and cultural heterogeneity play an important role. Under “full unification,” the rich region is the one that is prone to secede as it implicitly finances transfers to the poor region. The outcome will ultimately depend on the stakes at play for the rich region on the one hand and the poor region on the other hand. Put differently, whether “full union” or secession occurs at equilibrium rests on how much of their resources both regions are willing to put in the conflict to defend their own interests, which ultimately depends in the model on whether “full union” or secession is the socially efficient outcome. The author obtains mixed results as to whether fiscal decentralization may prevent wasteful secessionist conflicts. More specifically, it is shown that partial fiscal decentralization (which lies between the extreme cases of full unification and secession) may be successful to mitigate conflicts if the cost of diversity decreases proportionally with the level of decentralization and decentralization is a reversible process (should the later condition not been fulfilled, the rich region would be incited to spark a conflict in order to force secession once decentralization has been implemented). Finally, this issue should be address through empirics: Does decentralization tame or fuel pro-independence impulses?

22 Le Breton and Weber (2003) examine redistributive transfers from central to local governments to prevent secessions by the latter. They determine the structure of equalization transfers named partial equalization, which deter secession from disadvantaged and advantaged regions. Haimanko et al. (2004) extend the analysis of the trade-off between increasing return to scale and individual heterogeneity by combining both a game theory and operational research. They study the efficiency and the sustainability of population’s partition into several jurisdictions. Efficiency consists in minimizing total cost, while sustainability relies on secession-proof equilibriums. The authors establish that any efficient partition is sustainable in a unidimensional policy space. Drèze et al. (2008) highlight that this result does not hold in a two-dimensional policy space. They define the notion of *δ*-secession-proof allocation, where *δ* is the fraction of jurisdiction’s public good that is externally financed. This external transfer, which may be tiny but account for at least 0.2% of the total cost, is sufficient to ensure the stability of the partition.

23 Conversely, economies of scale mean that small newly independent countries would face high economic costs.

24 The authors use indistinctly the terms of autonomy, sovereignty, and self-determination.

25 Although this result seems intuitive, Perez-Sebastian and Raveh (2014) argue that economies dominated by natural resources are vulnerable to volatility of resource prices and to Dutch disease whereby periods of increase in prices translate into higher wages and general price levels in the economy, reducing competitiveness of the economy as a whole. Individuals in regions rich in natural resources may therefore prefer to remain in a union in order to pool the volatility-induced costs with the other regions. The authors do find that countries with unequal regional distribution of natural resources are more likely to be decentralized. This analysis, however, is not a direct test of the theory as it is not conducted at the group or region level.

26 Overall, richer groups are more likely to support self-determination movements than poorer ones. It is very likely that richer groups have a stronger bargaining position than poorer groups viz. the central government, which could explain why they are able negotiate satisfactory autonomy arrangement and thus avoid wars.

27 Ethnic civil wars represent roughly half of all civil wars since 1945 to 2005 (Wimmer et al. 2009).

28 The relationship between conflict and ethnicity is itself not obvious. As Fearon and Laitin (1996) note, most inter-ethnic interactions are peaceful. Yet, a recent body of evidence suggests that civil wars may well be more likely in heterogeneous settings. Montalvo and Reynal-Querol (2005) and Esteban et al. (2012) found that countries characterized by linguistic polarization are more prone to civil wars whereas Wimmer et al. (2009) found that participation in civil wars was more likely in countries linguistically fragmented. Denny and Walter (2014) provide a discussion for why civil wars may be more likely to be initiated by ethnic groups.

29 As explained by Wimmer et al. (2009, p. 317), “this data set records all politically relevant ethnic groups minorities and majorities, and their degree of access to executive-level state power – from total control of the government to overt political discrimination and exclusion. The EPR date set overcomes the limitations of existing data sets, especially the widely-used Minorities at risk data set, which focuses exclusively on disadvantaged minorities and is thus unable to capture the dynamics of ethnic politics at the power center”.

30 But the effect of decentralization is canceled or even reversed when indicators of bureaucratic quality are high. This is indicative of a complex interplay between decentralization and the strength of the state: whereas a well-functioning state (proxied by GDP per capita) is necessary for decentralized provision of public good to work, well-functioning states also have the means to be more predatory against ethnic minorities than weaker states (Arcand and Tranchant 2012).

31 Hug (2003) argues that since ethnic groups covered by MAR are selected on the basis of past violence and discriminations, the use of the dataset to study violence is potentially problematic. Studies based on the Ethno-Power Relations (EPR), which covers all politically-relevant ethnic groups in the world, should not suffer from selection bias.

## References

[CR1] Alesina A, Spolaore E (1997). On the number and size of nations. Quarterly Journal of Economics.

[CR2] Alesina A, Spolaore E, Wacziarg R (2000). Economic integration and political disintegration. American Economic Review.

[CR3] Alesina A, Spolaore E (2003). The size of the nations.

[CR4] Arcand, J.L. and Tranchant, J.P. (2012) 'Institutions, Mobilisation and Rebellion in Post-Colonial Societies', HiCN Working Paper 133, Brighton: Households in Conflict Network. http://www.hicn.org/wordpress/wp-content/uploads/2012/06/HiCN-WP-133.pdf..

[CR5] Arzaghi, M, & Henderson, VJ. (2005). Why countries are fiscally decentralizing. *Journal of Public Economics*, *89*(7), 1157–1189.

[CR6] Bakke, K, & Wibbels, E. (2006). Baltimore, Maryland: Diversity, disparity, and civil conflicts in federal states. *World Politics*, *59*(1), 1–50.

[CR7] Berkowitz D (1997). Regional income and secession: center-periphery relations in emerging market economies. Regional Science and Urban Economics.

[CR8] Bermeo NG (2002). The imports of institutions. Journal of Democracy.

[CR9] Bernheim BD, Peleg B, Whinston MD (1987). Coalition-proof equilibria I. Concepts. Journal of Economic Theory.

[CR10] Bolton, P, & Roland, G. (1997). The breakup of nations: a political economy analysis. *Quarterly Journal of Economics*, *112*(4), 1057–1090.

[CR11] Bolton, P, Roland, G, 1996, "Distributional conflicts, factor mobility, and political integration£", American Economic Review, Papers and Proceedings, LXXXVI, 99–104.

[CR12] Brancati D (2006). Decentralization: fueling the fire or dampening the flames of ethnic conflict. International Organization.

[CR13] Buchanan JM, Faith RL (1987). Secession and the limits of taxation: toward a theory of internal exit. American Economic Review.

[CR14] Bunce V (1999). Subversive institutions: the design and the destruction of socialism and the state.

[CR15] Casella, A., and Feinstein, JS. (2002). Public Goods in Trade: On the Formation of Markets and Jurisdictions. International Economic Review, 43(2), 437–462.

[CR16] Casella A (1994). Trade as engine of political change: a parable. Economica.

[CR17] Casella A (2001). The role of market size in the formation of jurisdiction. Review of Economic Studies.

[CR18] Cederman L-E, Hug S, Schadel A, Wucherpfennig J (2015). Territorial autonomy in the shadow of conflict: too little, too late?. American Political Science Review.

[CR19] Chandra K (2004). Why ethnic parties succeed? Patronage and ethnic counts in India.

[CR20] Christin T, Hug S (2012). Federalism, the geographic location of groups, and conflict. Conflict Management and Peace Science.

[CR21] Cohen FS (1997). Proportional versus majoritarian ethnic conflict management in democracies. Comparative Political Studies.

[CR22] Cornell SE (2002). Autonomy as a source of conflict: caucasian conflicts in theoretical perspective. World Politics.

[CR23] Cunningham K (2013). Understanding strategic choice: the determinants of civil war and nonviolent campaigns in self-determination disputes. Journal of Peace Research.

[CR24] Dafflon B, Madiès T (2011). L'économie politique de la décentralisation dans quatre pays d'Afrique sub-saharienne.

[CR25] Denny, K and Walter, B "Ethnicity and civil war", Journal of Peace Research, 51(2), 199–212.

[CR26] Desmet K, Breton M, Ortuño-Ortín I, Weber S (2011). The stability and breakup of nations: a quantitative analysis. Journal of Economic Growth.

[CR27] Dominicé, C. (2006). The Secession of the Canton of Jura in Switzerland. In M. Kohen (Ed.), Cambridge: Cambridge University Press. *Secession: International Law Perspectives*, 453–469. 10.1017/CBO9780511494215.016.

[CR28] Drèze J, Le Breton M, Savvateev S, Weber S (2008). Almost subsidy-free spatial pricing in a multi-dimensional setting. Journal of Economic Theory.

[CR29] Ellingsen T (1998). Externalities versus internalities: a model of political integration. Journal of Public Economics.

[CR30] Esteban, J, Mayoral, L, Ray, D. (2012). Ethnicity and Conflict: An Empirical Study. American Economic Review, *102*(4): 1310–1342.

[CR31] Fearon, J. and Laitin, DD. (1996). Explaining Interethnic Cooperation. American Political Science Review, 90(4), 715–735.

[CR32] Fearon JD, Laitin DD (1999). Weak states, rough terrain, and large-scale ethnic violence since 1945.

[CR33] Flamand, S., 2015. Partial decentralization as a way to prevent secessionist conflict. Unpublished Manuscript. http://sabineflamand.com/research/Conflict.pdf

[CR34] Gradstein M (2004). Political bargaining in a federation: Buchanan meets Coase. European Economic Review.

[CR35] Gurr TR (1994). Peoples against states: ethnopolitical conflict and the changing world system: 1994 presidential address. International Studies Quarterly.

[CR36] Haimanko O, Le Breton M, Weber S (2004). Voluntary formation of communities for the provision of public projects. Journal of Economic Theory.

[CR37] Hale HE (2004). Divided we stand: institutional sources of thnofederal state survival and collapse. World Politics.

[CR38] Hardgrave RL (1993). India: the dilemmas of diversity. Journal of Democracy.

[CR39] Hartmann, J. (2017). The Faroe Islands: possible lessons for Scotland in a new post-Brexit devolution settlement. *Edinburgh Law Review* forthcoming. https://papers.ssrn.com/sol3/papers.cfm?abstract_id=2909543

[CR40] Hechter M (2000). Containing nationalism.

[CR41] Hooghe L, Amoretti UM, Bermeo N (2004). Belgium: hollowing the center. Federalism and territorial cleavages.

[CR42] Horowitz D (1985). Ethnic Groups in Conflict.

[CR43] Hug S (2003). Selection bias in comparative research: the case of incomplete data sets. Political Analysis.

[CR44] Kymlicka W, Lehning PB (1998). Is federalism a viable alternative to secession?. Theories of secessionism.

[CR45] Laborderie V, Parent N (2012). Good morning Belgium.

[CR46] Le Breton M, Weber S (2003). The art of making everybody happy: how to prevent a secession.

[CR47] Lijphart A (1977). Democracies in plural societies: a comparative exploration.

[CR48] Martinez-Vazquez, J, Lago-Peñas, S, Sacchi, A. (2016). The impact of fiscal decentralization: a survey. *Journal of Economic Surveys* 31(4),1095-1129..

[CR49] Montalvo, José, G., and Marta Reynal-Querol. 2005. "Ethnic Polarization, Potential Conflict, and Civil Wars." American Economic Review, 95(3): 796–816.

[CR50] Nordquist K-A (1998). Autonomy as a conflict-solving mechanism: an overview.

[CR51] Oates WE (1972). Fiscal Federalism.

[CR52] Panizza U (1999). On the determinants of fiscal centralization: theory and evidence. Journal of Public Economics.

[CR53] Perez-Sebastian F, Raveh O (2014). Natural resources, decentralization, and risk sharing: can resource booms unify nations?.

[CR54] Persson T, Tabellini G (1996). Federal fiscal constitution: risk sharing and moral hazard. Econometrica.

[CR55] Reynaerts J, Vanschoonbeek J (2016). The economics of state fragmentation-assessing the economic impact of secession.

[CR56] Roeder PG (1991). Soviet federalism and ethnic mobilization. World Politics.

[CR57] Roethke, P. (2011). The right to secede under international law: the case of Somaliland. *Journal of International Service*, 38.

[CR58] Rosiere S (2010). La fragmentation de l’espace étatique mondial. L’Espace politique.

[CR59] Rota-Graziosi G (2004). La fragmentation politique, une revue de la littérature. Revue Française d’Economie.

[CR60] Rota-Graziosi G (2007). Secession and the limits of taxation: toward a theory of internal exit: comment. American Economic Review.

[CR61] Rousseau, D. (2014). L’équivoque référendaire. *La Vie des idées* 22 avril 2014. ISSN: 2105-3030. URL: www.laviedesidees.fr/L-equivoque-referendaire.html.

[CR62] Saideman S, Lanoue DJ, Campenni M, Stanton S (2002). Democratization, political institutions and ethnic conflict: a pooled time-series analysis, 1985-1998. Comparative Political Studies.

[CR63] Sambanis, N., M. Germann and A. Schädel, 2017. SDM: a new data set on self-determination movements with an application to the reputational theory of conflict. Journal of Conflict Resolution.

[CR64] Sambanis N, Milanovic B (2014). Explaining regional autonomy differences in decentralized countries. Comparative Political Studies.

[CR65] Snyder J (2000). From voting to violence: democratization and nationalist conflict.

[CR66] Sorens J (2005). The cross-sectional determinants of secessionism in advanced democracies. Comparative Political Studies.

[CR67] Spolaore, E (2010). Federalism, regional redistribution and country stability. In N Bosch, M Espasa, A Solé-Ollé (Eds.), *The political economy of inter-regional fiscal flows*. Cheltenham, UK and Northampton, Maryland: Edward Elgar Publishing.

[CR68] Suberu RT (2001). Federalism and ethnic conflict in Nigeria.

[CR69] Tancredi A, Cohen M (2006). A normative “due process” in the creation of states through secession. Secession: international law perspectives.

[CR70] Tiebout CM (1956). A pure theory of local expenditures. Journal of Political Economy.

[CR71] Toft MD (2003). The Geography of Ethnic Violence.

[CR72] Tranchant J-P (2008). Fiscal decentralization, institutional quality and ethnic conflict: a panel data analysis, 1985-2001. Conflict, Security and Development.

[CR73] Tranchant J-P (2016). Does autonomy of regional governments dampen ethnic conflict? A dynamic panel data analysis, 1950-2010.

[CR74] Van Hagen J, Eichengreen B (1996). Federalism, fiscal restraints, and European monetary union. American Economic Review.

[CR75] Walter B (2006). Information, Uncertainty, and the Decision to Secede. International Organization.

[CR76] Wimmer A, Cederman L-E, Min B (2009). Ethnic politics and armed conflict. A configurational analysis of a new global dataset. American Sociological Review.

[CR77] Zodrow GR, Mieszkovski P (1986). Pigou, Tiebout, property taxation, and the underprovision of local public goods. Journal of Urban Economics.

